# Photosensitive rash induced by nivolumab^☆^

**DOI:** 10.1016/j.abd.2021.07.007

**Published:** 2022-07-07

**Authors:** Francisco J. Navarro-Triviño, Ricardo Ruiz-Villaverde

**Affiliations:** a Department of Contact Eczema and Immunoallergic Diseases, Dermatology, Hospital Universitario San Cecilio, Granada, Spain; b Department of Dermatology, Hospital Universitario San Cecilio, Granada, Spain

**Keywords:** Melanoma, Patch tests, Photobiology

## Abstract

The therapeutic approach to metastatic melanoma has revolutionized the clinical course of this disease. Since 2011, different immunotherapeutic drugs have been approved. Nivolumab is a humanized immunoglobulin IgG4 monoclonal antibody that binds to the PD-1 receptor, blocking its interaction with his ligand PD-L1. The authors present a new case of photosensitivity induced by nivolumab. The photo exposed distribution of the eruption, the sun exposure prior to the beginning of the eruption, and the chronological relationship with the beginning of the treatment are data that have allowed us to confirm the suspected clinical diagnosis.

## Case report

**Fig. 1:**
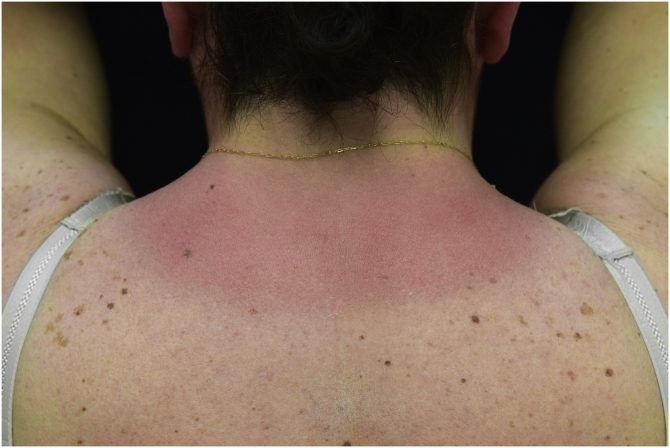
Erythematous rash located in photo-exposed areas: neck.

**Fig. 2:**
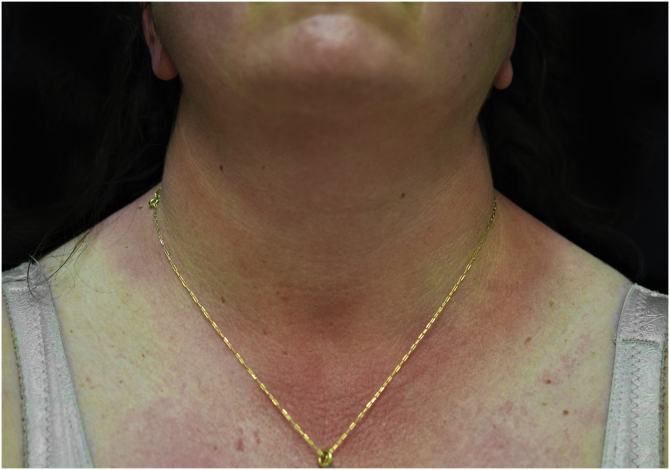
Erythematous rash located in photo-exposed areas: neckline.

**Fig. 3:**
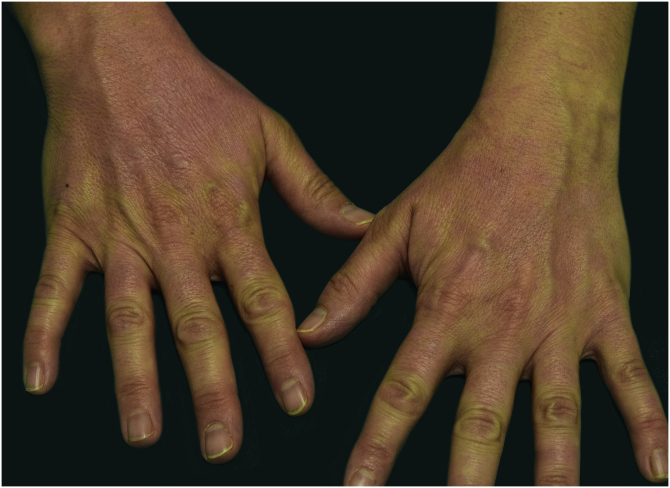
Erythematous rash located in photo-exposed areas: back on hands.

**Fig. 4:**
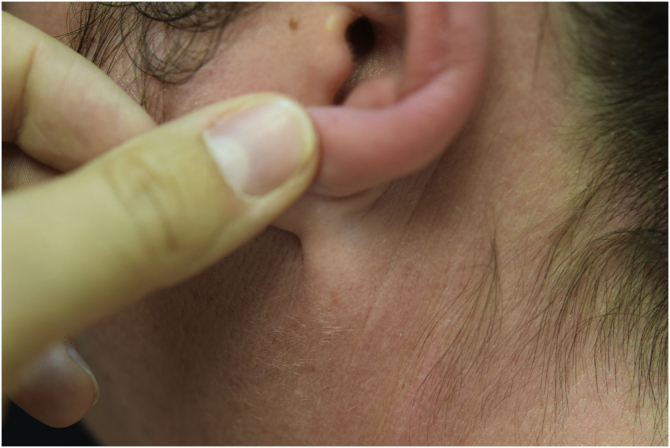
Erythematous rash located in photo-exposed areas: earlobe fold is respected.

**Fig. 5:**
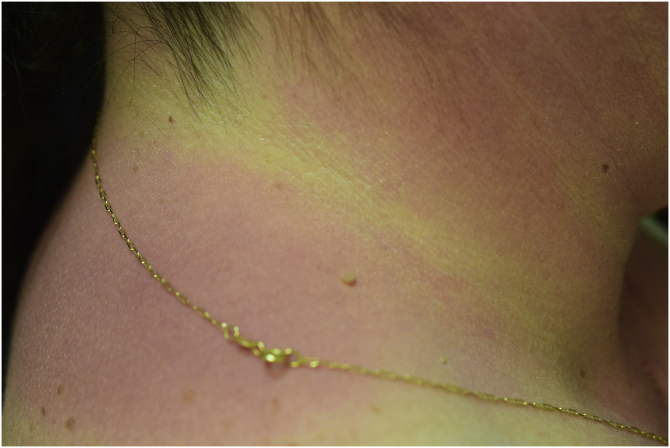
Erythematous rash located in photo-exposed areas: choker area is respected.

A 44-year-old woman with a diagnosis of metastatic melanoma was referred to the present study’s dermatology department due to a 1-week evolution of cutaneous rash. She had started treatment with nivolumab 3-months ago. The patient did not refer to the consumption of any other medication. On her medical history, no previous urticaria or cutaneous rash had been noted. Intermittent sun exposure prior to the pruritic skin rash was reported. Physical examination revealed an erythematous eruption located on photo-exposed areas such as the neck, neckline, and back of the hands (Fig. 1, Fig. 2, Fig. 3). The crease of the earlobe and the area of the neck covered by the choker were respected (Fig. 4, Fig. 5). The patient denied performing a skin biopsy. Treatment with oral antihistamine (cetirizine 10 mg/day) and topical corticosteroid (mometasone furoate 0.1% cream once/day) was indicated until the resolution of the rash. The use of photoprotective clothing and a topical photoprotector covering the 3 radiation spectra (Intense Protect, Avene®) was indicated. Complete improvement was observed at the 2-weeks follow-up. Photo-test showed a Decreased Minimal Erythema (DME) dose of 4 J/cm^2^ UVA (Ultraviolet-A), without showing changes in the DME for UVB (Ultraviolet-B) or visible light. The laboratory test ruled out the presence of autoantibodies and anomalies on complement or immunoglobulin levels. Nivolumab was not stopped in consensus with the Oncology department. Currently, the patient is undergoing periodic check-ups using sunscreen clothing and cream without changes for 3-months.

## Discussion

Anti-PD-1 antibody immunotherapies, pembrolizumab, and nivolumab, are drugs approved for the treatment of metastatic melanoma. These drugs bind PD-1 receptors, thus blocking attachment to PD-L1 (Programmed Death-ligand 1) and PD-L2 (Programmed Death-ligand 2), releasing PD-1 pathway inhibition. Adverse Effects (AEs) are dose-related and reversible.

Nivolumab is a humanized immunoglobulin IgG4 monoclonal antibody that binds to the PD-1 receptor, blocking its interaction with its ligand PD-L1. Pruritus, rash, vitiligo, maculopapular rash, and dry skin stand out among the most frequent dermatological Adverse Effects (AE) that nivolumab can produce in monotherapy treatment. Most of these adverse effects appear in the first 5-weeks of treatment and are independent of whether the patient has received ipilimumab as prior therapy^1^.

Photosensitivity has been described with BRAF inhibitors, mainly vemurafenib, and anti-CTLA4 ipilimumab. A single case of photosensitivity has been reported in a patient receiving the nivolumab/ipilimumab combination to date^2^, although in the longest series published to date, which includes 576 patients receiving nivolumab therapy, 8 patients with grades 1 and 2 photosensitivity reaction have been described, without further mentioning in terms of outcome and treatment.

Several cases of subacute cutaneous lupus induced by nivolumab have also been reported^3^, ^4^. Ciccolini et al.^5^ has reported recently a systematic review regarding the risk of photosensitivity with immunotherapy against melanoma. Vemurafenib and vandetanib have been communicated as the main therapies with a higher risk of photosensitivity. The report of photosensitive dermatoses induced by the drug requires proper educational measures for these patients. Photoprotection and adequate sun exposure will be crucial to avoid these adverse skin effects.

## Financial support

None declared.

## Authors’ contributions

Francisco J. Navarro-Triviño and Ricardo Ruiz-Villaverde: Approval of the final version of the manuscript; critical literature review; data collection, analysis and interpretation; effective participation in research orientation; intellectual participation in propaedeutic and/or therapeutic management of studied cases; manuscript critical review; preparation and writing of the manuscript; statistical analysis; study conception and planning.

## Conflicts of interest

None declared.
